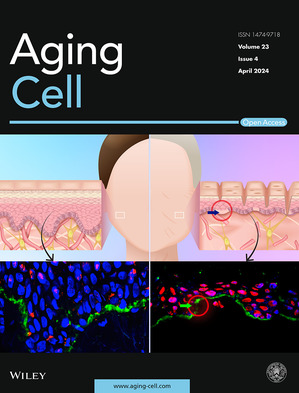# Additional Cover

**DOI:** 10.1111/acel.14180

**Published:** 2024-04-15

**Authors:** Eva Roig‐Rosello, Guila Dayan, Simone Bovio, Patricia Manissier, Elisabeth Errazuriz, Patricia Rousselle

## Abstract

Cover legend: The cover image is based on the Research Article *Dermal stiffness governs the topography of the epidermis and the underlying basement membrane in young and old human skin* by Eva Roig‐Rosello et al., https://doi.org/10.1111/acel.14096.